# Key Targets and Molecular Mechanisms of the Fat-soluble Components of Ginseng for Lung Cancer Treatment

**DOI:** 10.1007/s12010-023-04409-w

**Published:** 2023-03-04

**Authors:** Dongdong Gao, Yingyue Li, Sen Xiang, Jing Zhang

**Affiliations:** 1 Department of Oncology, Zhumadian Central Hospital, 463000 Zhumadian, China; 2 Medical Engineering Technology and Data Mining Institute, Zhengzhou University, 450001 Zhengzhou, China; 3 School of Medicine, Huanghuai University, 463000 Zhumadian, China

**Keywords:** Ginseng, Fat-soluble components, Lung cancer, Network pharmacology, Regulatory mechanism, Pathway enrichment analysis

## Abstract

Objective: To analyze the regulatory effects and key targets of the fat-soluble components of ginseng in lung cancer. Methods: Gas chromatography–mass spectrometry and the Traditional Chinese Medicine Systems Pharmacology Database and Analysis Platform were used to analyze and identify the fat-soluble components of ginseng. Network pharmacology was used to analyze the therapeutic targets of the fat-soluble components of ginseng in lung cancer and screen key proteins. In vitro assays were conducted to verify the effects of the active fat-soluble components of ginseng on proliferation and apoptosis in lung cancer cells and to verify the regulation of key proteins. Results: Ten active fat-soluble components of ginseng were screened for follow-up. Network pharmacology showed 33 overlapping targets between the active fat-soluble components of ginseng and lung cancer, and functional enrichment of the targets showed involvement of response to nitrogen, hormone response, membrane raft, and positive regulation of external stimulus. Pathway enrichment analysis showed vascular endothelial growth factor (VEGF) signaling, adipocyte lipolysis regulation, chronic myelogenous leukemia, endocrine resistance, and NSCLC-related pathways. A protein-protein interaction network was constructed, and the top 10 targets were selected in accordance with their scores. Ultimately, five target genes (EGFR, KDR, MAPK3, PTPN11, and CTNNB1) were selected in combination with literature mining for subsequent experimental verification. Proliferation assays showed that the growth of lung cancer cells was significantly decreased in a concentration-dependent manner in the fat-soluble components of ginseng intervention group compared with controls. Flow cytometry showed that active fat-soluble components of ginseng promoted apoptosis in a concentration-dependent manner in lung cancer cells. Western blot and quantitative real-time PCR showed that levels of the five key proteins and mRNAs were significantly decreased in the intervention group; furthermore, histone protein and mRNA levels were significantly higher in the high-concentration intervention group compared with the low-concentration group. Conclusion: The active fat-soluble components of ginseng inhibited the growth of lung cancer cells and promoted apoptosis. The underlying regulatory mechanisms may be related to signaling pathways involving EGFR, KDR, MAPK3, PTPN11, and CTNNB1.

## Introduction

Lung cancer is a carcinoma in situ that originates from bronchial epithelial cells and is the leading cause of cancer-related deaths worldwide [1, 2]. Clinical treatments for lung cancer include surgery, chemotherapy, radiotherapy, targeted therapy, and other comprehensive treatments. However, the median survival time for advanced non-small cell lung cancer (NSCLC) patients is only 8 to 10 months [3], and the median survival time of patients with small cell lung cancer is 11 months [4]. Traditional Chinese medicine (TCM) has also recently played positive roles in reducing rates of recurrence and metastasis, alleviating side effects of radiotherapy, chemotherapy, and targeted therapy, and improving the quality of life of lung cancer patients.

As a commonly used Chinese medicinal herb, ginseng has the functions of invigorating vitality, reactivating veins, tonifying the spleen, and benefiting the lungs. Several studies have shown that ginseng can be beneficial for lung cancer treatment by inhibiting cell proliferation, inducing cell cycle arrest, promoting apoptosis, and affecting cell differentiation [5–7]. However, due to the complex composition of ginseng, the pharmacological mechanisms of its activity in lung cancer remain unclear. Network pharmacology is an emerging discipline to discover new drugs, drug efficacy, and mechanisms of action from the perspective of systems biology and biological networks on the basis of high-throughput omics data analysis, virtual computing, and network database retrieval [8]. This study was based on the research idea of the multi-component and multi-target effects of TCM; thus, network pharmacology was applied to screen key targets and construct a “component-target-disease-pathway” protein-protein interaction (PPI) network by analyzing the targets, biological functions, and pathways of the active fat-soluble components of ginseng for lung cancer treatment. Key targets were further verified by in vitro experiments to elucidate the biological mechanisms of the active fat-soluble components of ginseng and provide new ideas and directions for treating lung cancer.

## Materials and Methods

### Detecting the Fat-soluble Components of Ginseng

Ginseng (30 g) was crushed and filtered, evenly divided into three portions of 10 g each, and then ethanol, methanol, and benzenol were used for hot water extraction for 4 h. The resulting filtrates were concentrated to 5 mL by rotary evaporation after ultrafiltration through a 0.22 μm filter membrane. The fat-soluble components were then dissolved in ethyl ether, and 1.5 mL of each was taken for gas chromatography–mass spectrometry (GC-MS) analysis.

1. *Gas chromatography conditions*: RTX-5MS (30 m × 0.25 mm, 0.25 μm) chromatographic elastic quartz capillary columns were used. A temperature program was adopted, with an original column temperature of 180 °C. This was raised to 210 °C at 10 °C/min, then to 220 °C at 1 °C/min, and then to 280 °C at 10 °C/min, which was held for 1 min; the total measurement time was 20 min. The split ratio was set to 20:1. The temperature was 280 °C. The pressure was 50 KPa. The injection volume was 1 µL, with high-purity (> 99.9%) nitrogen as the medium. The flow rate was controlled at 1.1 mL/min.
2. *Mass spectrometry conditions*: The ion source was EI (70 eV). The temperature was 200 °C. The connector temperature was 250 °C. The solvent delay parameter was 3.5 min. The scanning area was 50 to 600 amu. The voltage of the electron multiplier was 1200 V.
3. *Literature screening*: Identified components were screened in accordance with the generated compound ion flow diagram and by referring to relevant literature, comparing base peaks, mass-to-charge ratios, relative abundance and other parameters; furthermore, chemical compositions, physical and chemical properties, and pharmacological effects were determined.

### Screening and Identifying the Fat-soluble Components of Ginseng

To further define the set of active fat-soluble components in ginseng the Traditional Chinese Medicine Systems Pharmacology Database and Analysis Platform (TCMSP) was used to screen the active fat-soluble components of ginseng detected by GC-MS, with the following conditions: oral bioavailability (OB) ≥ 30% and drug-like property (DL) ≥ 0.1.

### Predicting Effects of the Fat-soluble Components of Ginseng on Human Targets

Target genes related to the fat-soluble components of ginseng were predicted using the SwissTargetPrediction tool (http://www.swisstargetprediction.ch/), which was performed using *Homo sapiens* as the organism, entering the simplified molecular-input line-entry system of the main chemical composition, and setting the probability > 0.1 as the screening condition. The Bioinformatics Analysis Tool for Molecular Mechanisms of Traditional Chinese Medicine (BATMAN-TCM, http://bionet.ncpsb.org/batman-tcm/) tool was used for target prediction of ginseng fat-soluble components, with the parameters of score = 20, P-value = 0.05.

### Target Analysis in Lung Cancer

The dataset of differentially expressed genes in lung cancer (GSE176348) was screened from the Gene Expression Omnibus (GEO) database using the GEO2R tool. A Venn diagram (http://bioinformatics.psb.ugent.be/webtools/Venn/) was drawn to screen the overlapping genes of lung cancer targets and potential targets of ginseng fat-soluble components. Potential targets of ginseng fat-soluble components with roles in lung cancer were used for subsequent analysis.

### Gene Ontology (GO) and Kyoto Encyclopedia of Genes and Genomes (KEGG) Enrichment Analysis

The Metascape online tool (http://metascape.org/) was used for the enrichment analysis of potential targets of ginseng fat-soluble components, including GO and KEGG enrichment analysis.

### Construction of Regulatory Networks

A ginseng fat-soluble components–target regulation network, a TCM–component–target–disease network, and a TCM–component–target–pathway network were constructed using Cytoscape 3.7.1.

### Construction of the PPI

In this study, the PPI network of potential targets of ginseng fat-soluble components in lung cancer was constructed primarily using the STRING database combined with the Cytoscape tool. The top 10 targets of the core were screened using the Cytohubba plug-in.

### Experimental Verification

1. *Cell culture and treatment*: The human lung cancer cell line NCI-H1299 was purchased from Shenyang Wan Shi Bio (Shenyang, China) and cultured in RMI1640 medium containing 20% fetal bovine serum at 37 °C in 5% CO_2_. NCI-H1299 cells cultured to logarithmic growth phase were randomly divided into six groups, which were treated with different doses of ginseng fat-soluble components (0, 50, 100, 150, 200, and 250 µg/mL). Cells from each group were collected 48 h after treatment for subsequent analysis.
2. *Cell counting kit-8 (CCK-8) assay*: Cells of each group were plated in 100 µL of complete medium into 96-well plates, and then incubated with 10 µL of CCK-8 solution at 37 °C for 1 h. OD was measured at 450 nm on a microplate analyzer.
3. *Flow cytometry*: Apoptosis was detected by Annexin V-FITC/propidium iodide (PI) staining and cell sorting. Briefly, cells in each group were collected by centrifugation, washed twice with phosphate buffer saline, and resuspended in 500 µL binding buffer. Next, the cells were sequentially incubated with 5 µL Annexin V-FITC, and then with 10 µL PI in the dark for 5 to 15 min. The results were detected by flow cytometry.
4. *Western blot analysis*: Total protein was extracted in 100 µL RIPA lysis buffer supplemented with 1 µL PMSF, 1 µL phosphatase inhibitor, and 1 µL protease inhibitor. Protein quantification was performed using the BCA protein quantification kit in accordance with manufacturer’s instructions. Samples were mixed at a 4:1 ratio with 5× loading buffer, boiled for 10 min, and stored at − 20 °C. Equal amounts of protein were separated by 10% sodium dodecyl sulfate polyacrylamide gel electrophoresis, and then transferred to membranes after electrophoresis. The membranes were blocked in 5% nonfat milk-tris buffered saline tween at room temperature for 2 h, washed three times with TBST, and then incubated overnight at 4 °C with the following primary antibodies: anti-EGFR, anti-KDR, anti-MAPK3, anti-PTPN11, anti-CTNNB1 (all diluted 1:1000), and anti-GAPDH (diluted 1:10000). Next, the membranes were washed three times with TBST and incubated with HRP-labeled goat anti-rabbit IgG or HRP-labeled goat anti-mouse IgG (both diluted 1:10000) secondary antibodies at room temperature for 2 h. Immunoreactive bands were then visualized by enhanced chemiluminescence rendering. Films were developed and scanned, and the optical density values of target bands were analyzed using Gel-Pro-Analyzer software.
5. *Quantitative real-time PCR (qPCR)*: Total RNA was extracted from cells by the TRIzol-chloroform-isopentyl alcohol method (Thermo Fisher Scientific, Waltham, MA, USA), and cDNA was synthesized using a reverse transcription kit (Servicebio, Wuhan, China). The relative expression levels of the indicated genes were detected by qPCR. Primer sequences are shown in Table 1. PCR reactions comprised 2 × qPCR Mix (12.5 µL), 2.5 µM primers (0.5 µL), reverse transcription product (2.0 µL), and ddH_2_O (4.0µL). The qPCR conditions were 95 °C for 10 min, and then 40 cycles of 95 °C for 15 s, 62 °C for 30 s, and 72 °C for 30 s; the melting curve was from 60 to 95 °C, heating 0.5 °C every 15 s. The 2^−ΔΔCt^ method was used to calculate relative mRNA expression, with β-actin as the internal reference.

**Table 1:** List of primer sequences (5′–3′)

Name	Primer sequence
homo CTNNB1 F	CTGCCAAGTGGGTGGTATA
homo CTNNB1 R	GGGATGGTGGGTGTAAGAG
homo KDR F	AATAATCAGAGTGGCAGTG
homo KDR R	ACATAAATGACCGAGGC
homo MAPK3 F	GGGAGGTGGAGATGGTG
homo MAPK3 R	GCTGGCAGTAGGTCTGATGT
homo PTPN11 F	AGAGGAGTTGATGGCAGTT
homo PTPN11 R	TTCTGAATCTTGATGTGGG
homo EGFR F	CATCTCCGAAAGCCAACA
homo EGFR R	CGACGGTCCTCCAAGTAG
homo β-actin F	GGCACCCAGCACAATGAA
homo β-actin R	TAGAAGCATTTGCGGTGG

### Statistical Analysis

Experimental data were analyzed and plotted using SPSS 23.0 (IBM, Armonk, NY, USA) and GraphPad Prism 5.0 (GraphPad Software, Inc., San Diego, CA, USA). Comparisons of measurement data were performed by the Student’s t test, with P < 0.05 used to indicate significant differences.

## Results

### Identifying the Fat-soluble Components of Ginseng by GC-MS

**Fig. 1:**
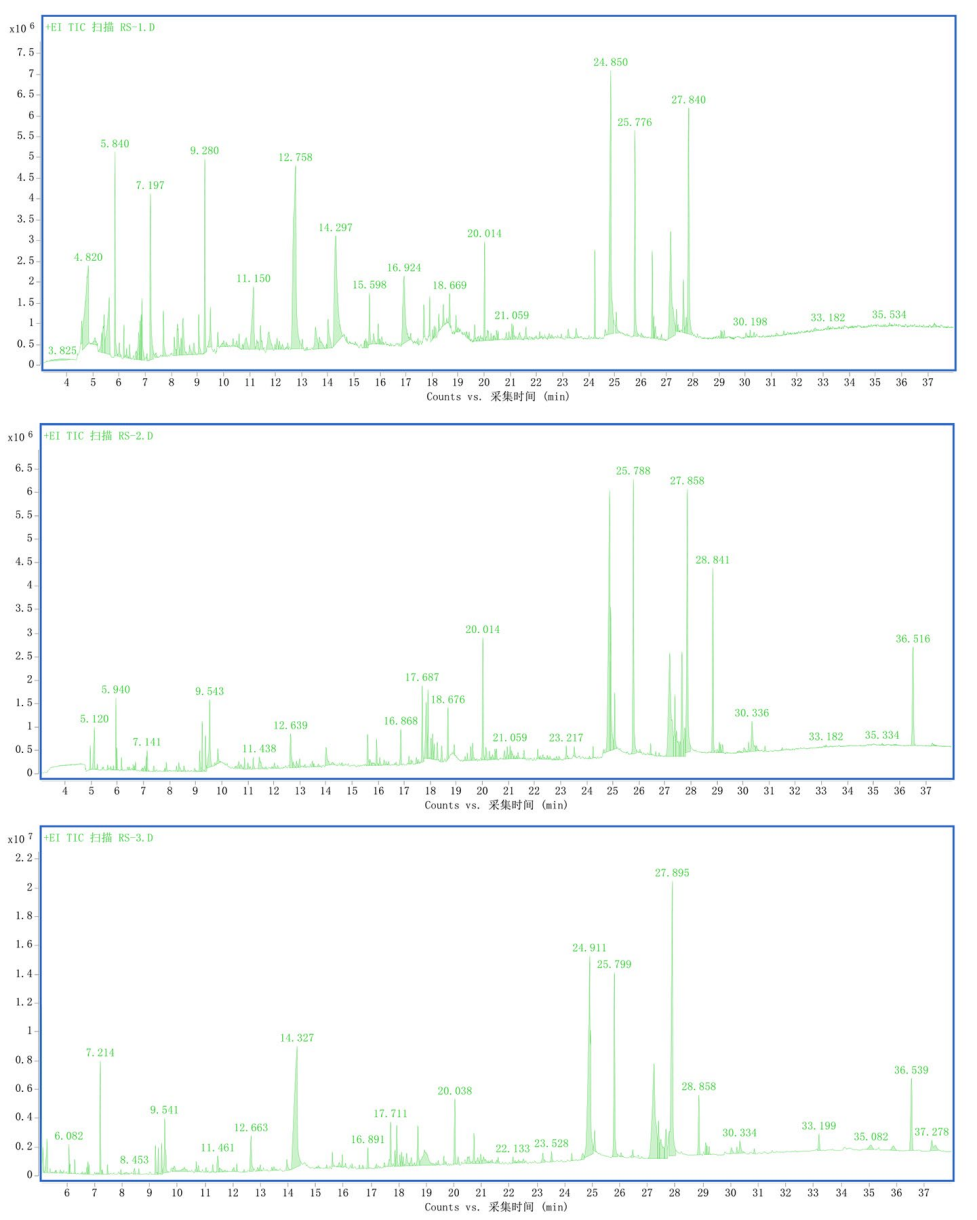
Gas chromatography–mass spectrometry ion chromatograms of ginseng extracts prepared using the (A) methanol, (B) ethanol, and (C) benzene alcohol extraction methods

Components of ginseng were extracted by the methanol, ethanol, and phenyl alcohol extraction methods and then analyzed by GC-MS. The ion chromatogram results are shown in Fig. 1. The main fat-soluble components of ginseng were obtained by ion chromatography. At the same time, the main fat-soluble active ingredients of ginseng in the TCMSP database, under the screening conditions OB ≥ 30% and DL ≥ 0.1 were as follows: 8,11-octadecadienoic acid methyl ester, Oleicacid, Linoleicacid, Panaxydol, Phenylglucoside, 1 H-Cycloprop[e]azulen-7-ol, decahydro-1,1,7- trimethyl-4-methylene-,[1ar-(1a.α,4a.α,7.β,7a.β,7b.α)]-, Calarene, (S,Z)-heptadeca- 1,9-dien-4,6-diyn-3-ol, Palmitoleic acid, and (+)-Ledene (Table 2).

**Table 2:** The main fat-soluble components of ginseng extract

Molecule name	OB(%)	DL	PubChem CID
8,11-Octadecadienoicacid methylester	41.93	0.17	5,319,737
Oleicacid	33.13	0.14	445,639
Linoleicacid	41.9	0.14	5,280,450
Panaxydol	61.67	0.13	126,312
Phenylglucoside	57.42	0.12	65,080
1 H-Cycloprop[e]azulen-7-ol,decahydro-1,1,7-trimethyl-4-methylene-,[1ar-(1a.α,4a.α,7.β,7a.β,7b.α)]-	82.33	0.12	6,432,640
Calarene	52.16	0.11	28,481
(S,Z)-Heptadeca-1,9-dien-4,6-diyn-3-ol	43.31	0.1	5,469,789
Palmitoleic acid	35.78	0.1	445,638
(+)-Ledene 1 H-Cycloprop[e]azulene,1a,2,3,5,6,7,7a,7b-octahydro-1,1,4,7-tetramethyl-,[1aR-(1a.α,7.α,7a.β,7b.α)]-	51.84	0.1	10,910,653

### Identification of Potential Targets of Ginseng Fat-soluble Components in Lung Cancer

**Fig. 2:**
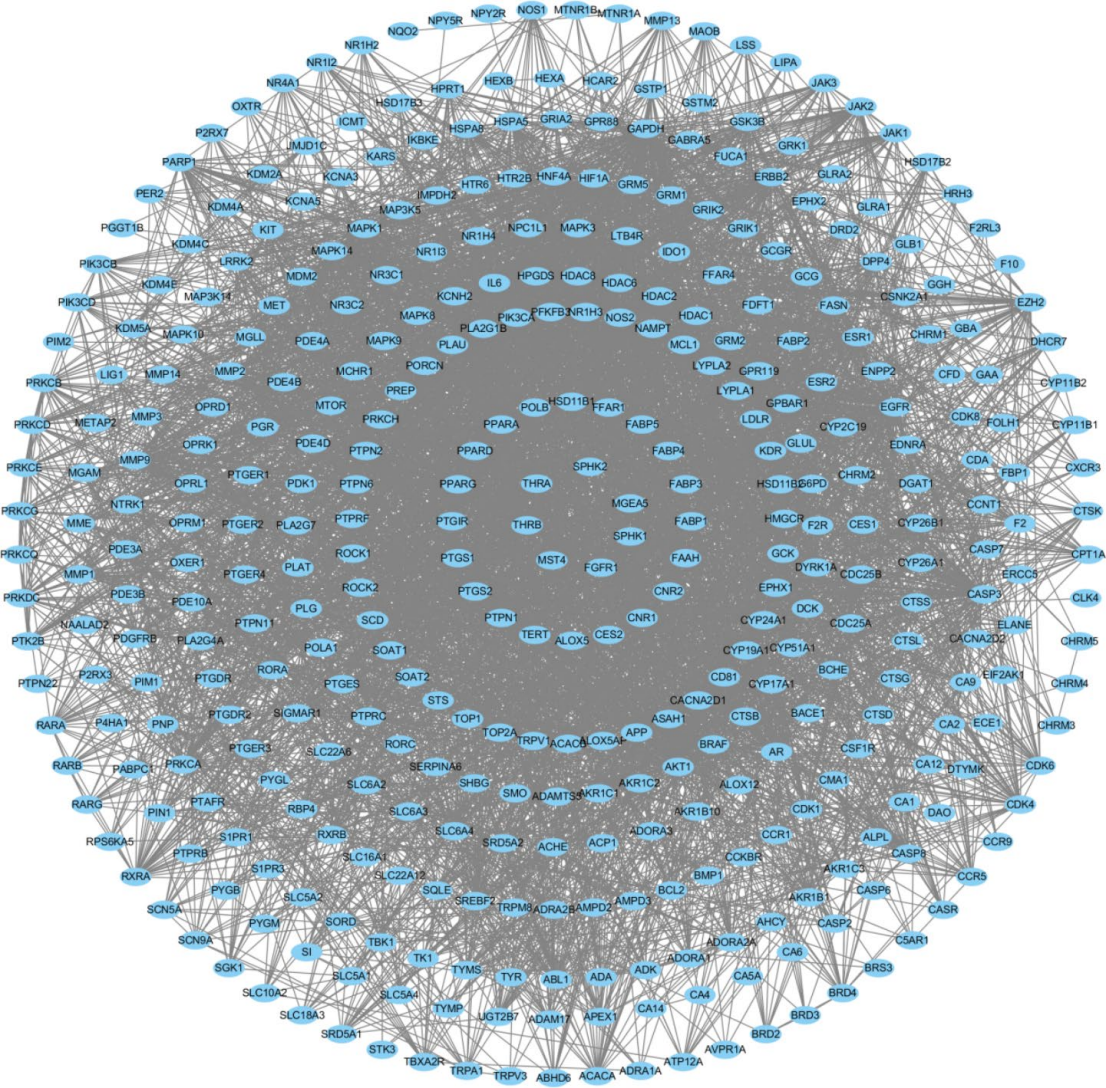
Gene targets corresponding to the active fat-soluble components of ginseng

**Fig. 3:**
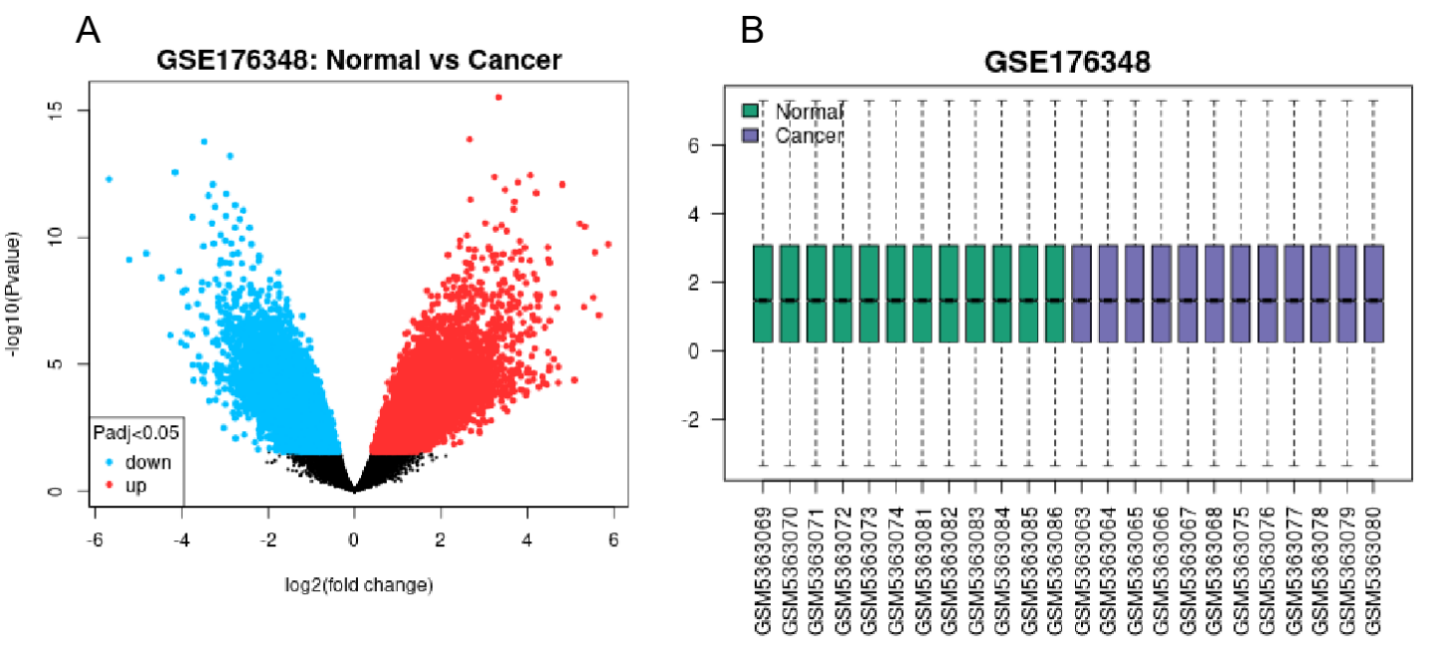
Volcano plot of differentially expressed genes in lung cancer; (A) the volcano plot; (B) normalized map from the analyzed specimens

**Fig. 4:**
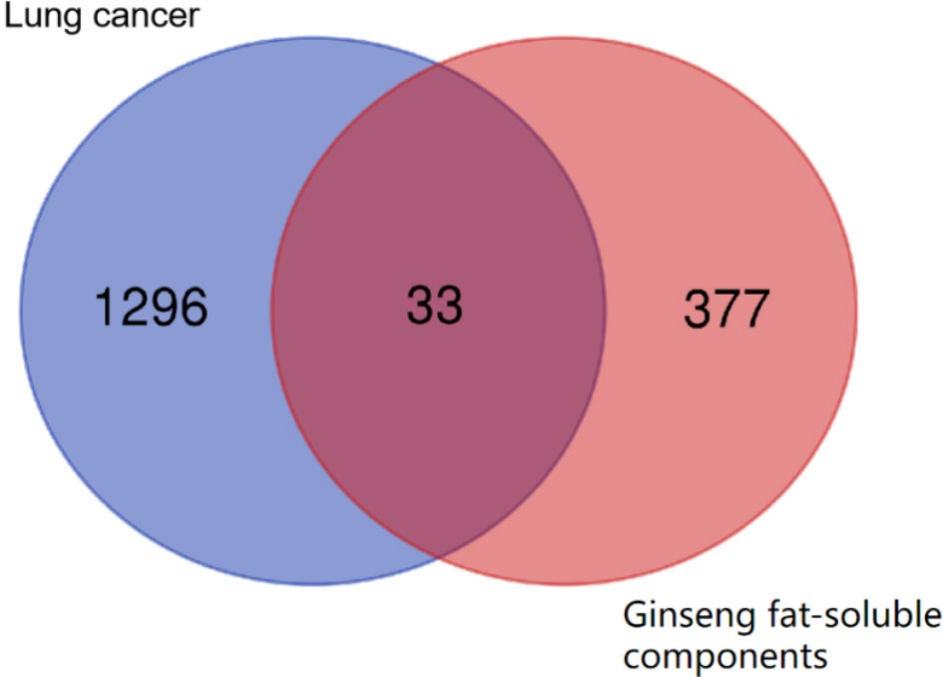
Venn diagram of potential lung cancer targets of ginseng fat-soluble components

SwissTargetPrediction and BATMAN-TCM were used to analyze 10 fat-soluble components of ginseng extract, from which 410 targets were obtained (Fig. 2). The GSE176348 dataset from the GEO database was screened for differentially expressed genes using the GEO2R tool. The volcano plot results are shown in Fig. 3. The GSE176348 dataset included 12,291 differentially expressed genes for lung cancer, including 5,859 up- and 6,432 down-regulated genes. To narrow the results, 1329 differentially expressed genes in lung cancer with |log2| ≥2 were selected. Next, the 410 gene targets screened by the active fat-soluble components of ginseng and 1329 differentially expressed genes in lung cancer were compared using Venny 2.1.0 to obtain the relationship between ginseng fat-soluble components and lung cancer. The Venn diagram with 33 common targets is shown in Fig. 4.

### Enrichment Analysis of Key Targets of Ginseng Fat-soluble Components for Lung Cancer Intervention

**Fig. 5:**
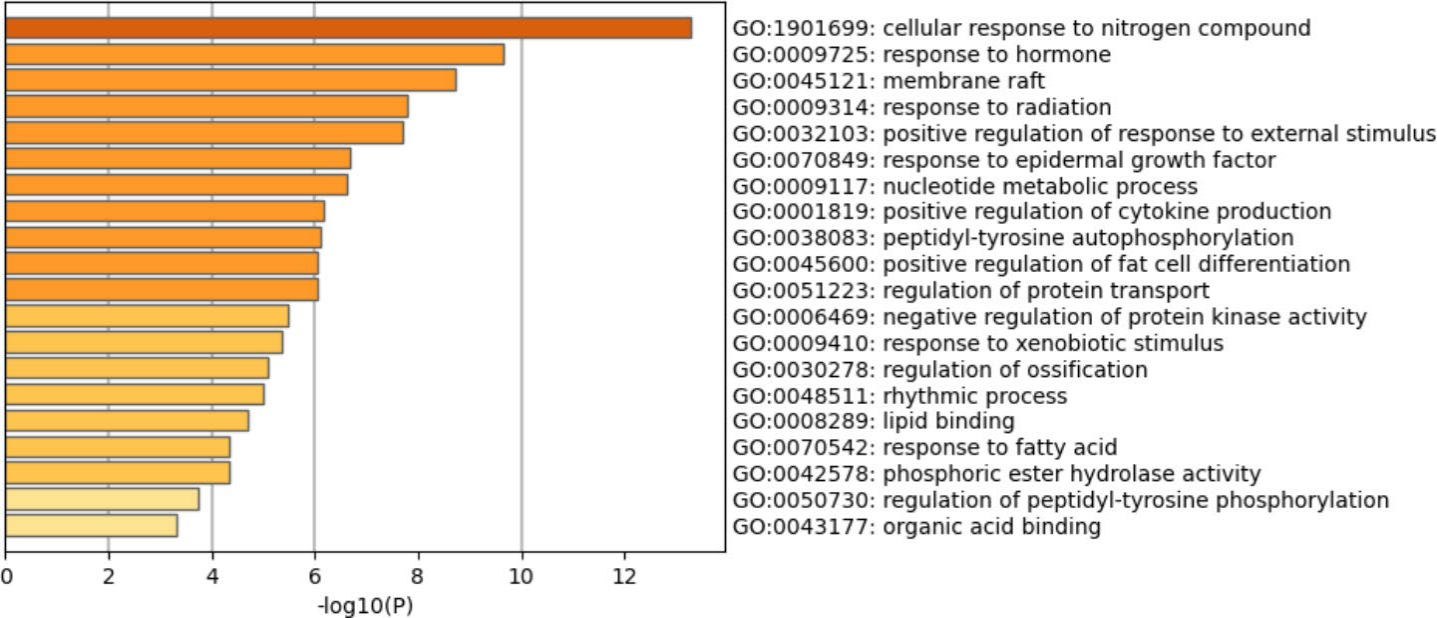
Gene Ontology analysis of key targets of ginseng fat-soluble components in lung cancer

**Fig. 6:**
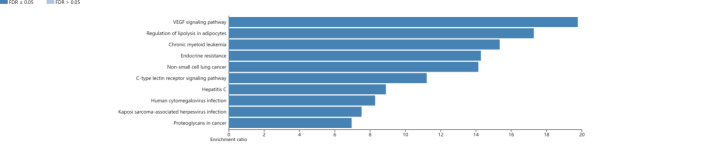
Kyoto Encyclopedia of Genes and Genomes analysis of key targets of ginseng fat-soluble components in lung cancer

Functional enrichment analysis and pathway enrichment analysis were performed on the key targets of the effective fat-soluble components of ginseng for lung cancer treatment, and the results are shown in Figs. 5 and 6. The top five hits in the functional enrichment analysis were response to nitrogen, hormone response, membrane raft, radiation response, and positive regulation of external stimulus. The top five pathways in the pathway enrichment analysis were VEGF signaling, adipocyte lipolysis regulation, chronic myelogenous leukemia, endocrine resistance, and NSCLC.

### Analysis and Network Construction of Key Targets of Ginseng Fat-soluble Components for Lung Cancer Intervention

**Fig. 7:**
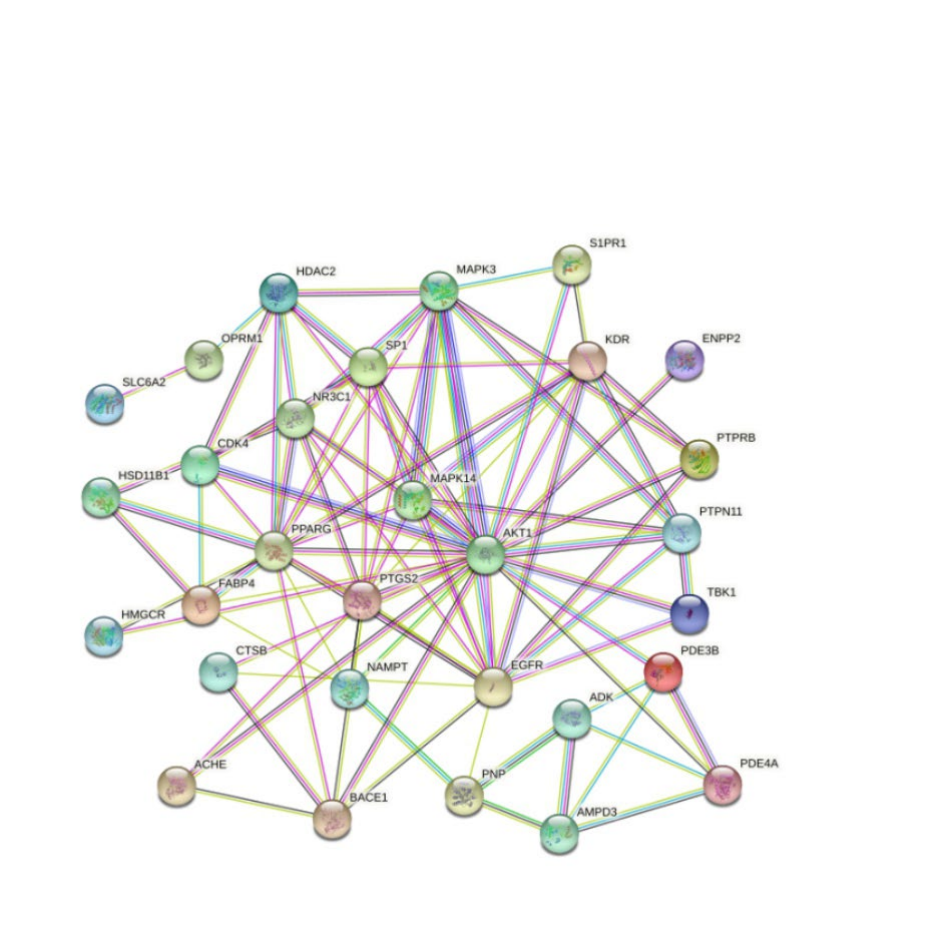
Protein-protein interaction network of the key targets of ginseng fat-soluble components for lung cancer intervention

**Fig. 8:**
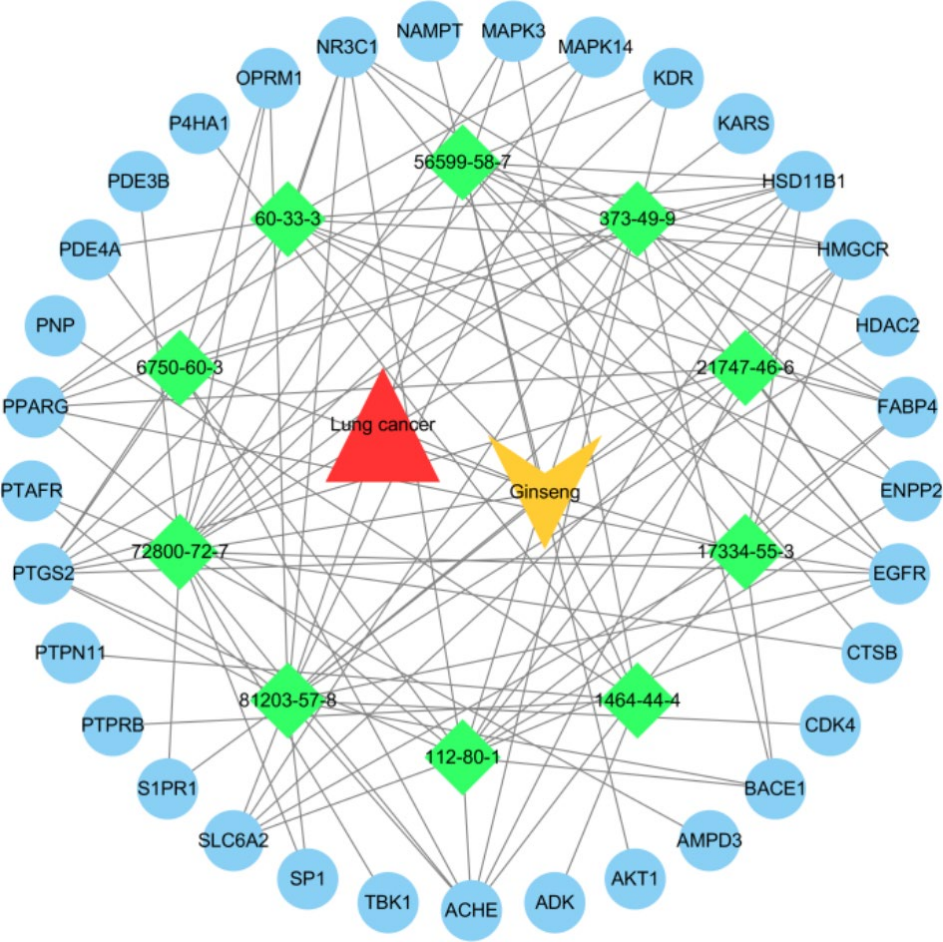
Traditional Chinese medicine-component-target-disease network diagram (the triangle is lung cancer, diamonds are the 10 active fat-soluble components of ginseng, the V-shape is ginseng, and circles are targets)

**Fig. 9:**
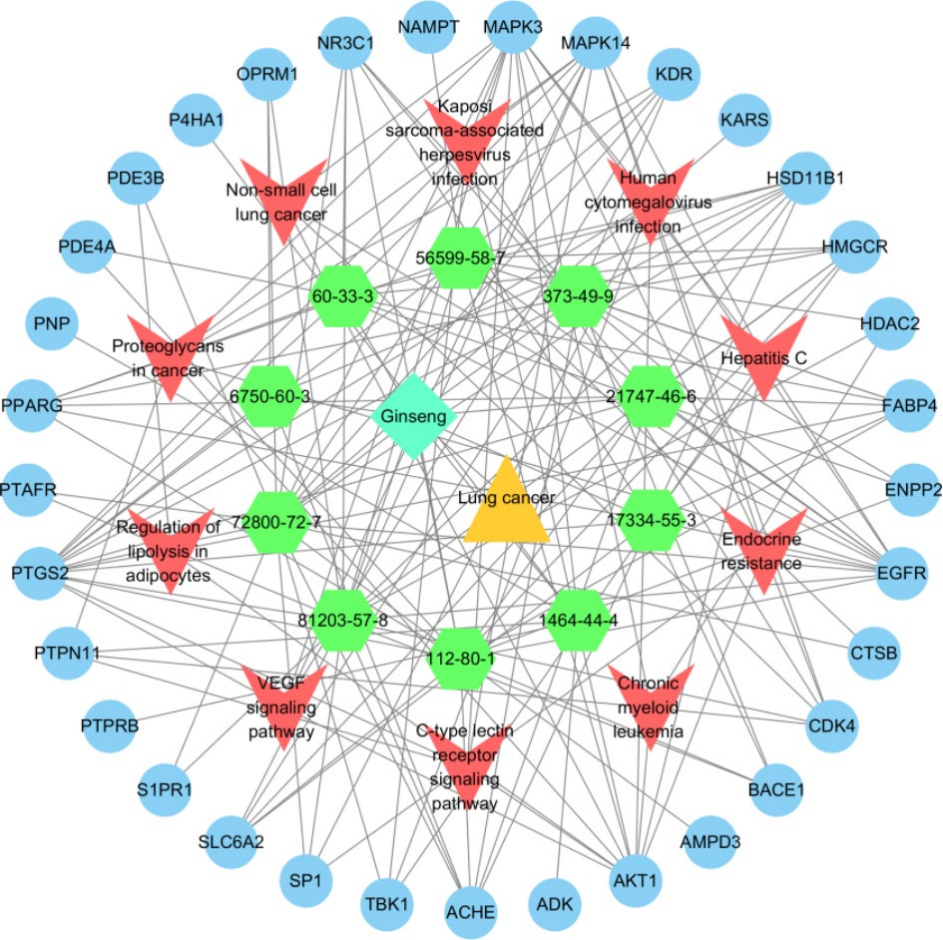
Traditional Chinese medicine-component-target-pathway network diagram (the rhombus is ginseng, the triangle is lung cancer, hexagons are the 10 active fat-soluble components of ginseng, V-shapes are pathways, and circles are targets)

**Fig. 10:**
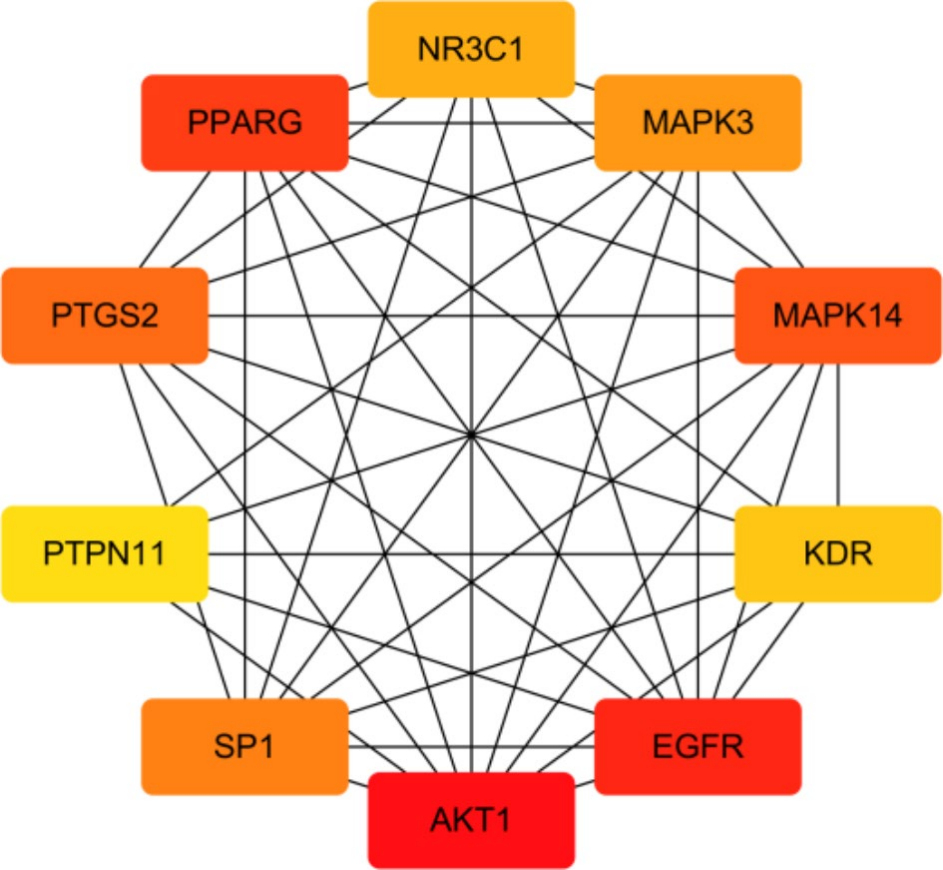
Top 10 targets of ginseng fat-soluble components for lung cancer intervention

A PPI network of 33 key targets of the active fat-soluble components of ginseng for lung cancer intervention was constructed using the STRING database (Fig. 7). We further constructed a TCM-component-disease-target network (Fig. 8) and a TCM-component-pathway-target network (Fig. 9) using Cytoscape. By combining literature mining with the 33 key targets and the top 10 screening targets, we chose EGFR, KDR, MAPK3, PTPN11, and CTNNB1 for subsequent molecular experiments (Fig. 10).

### Toxicological Effects of Ginseng Fat-soluble Components on Lung Cancer Cells

**Fig. 11:**
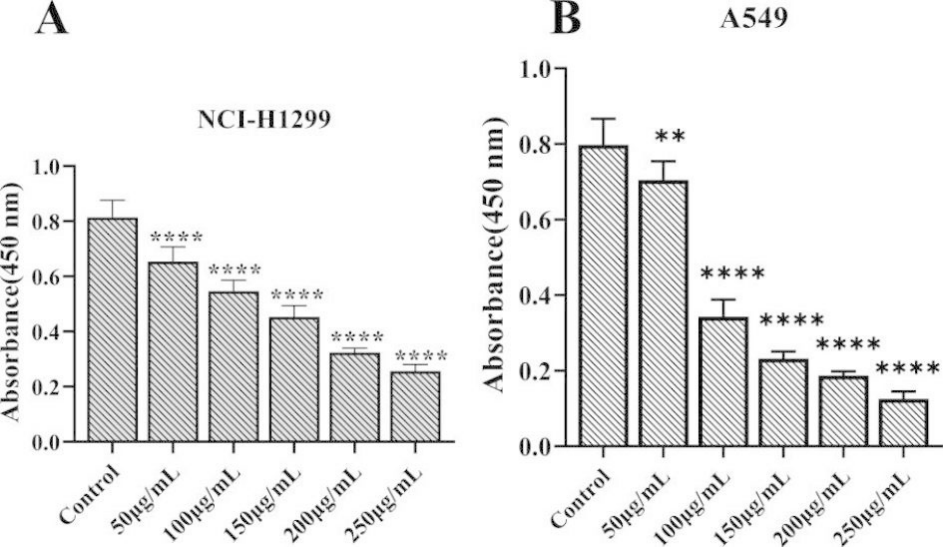
Inhibitory effects of ginseng fat-soluble components at different concentrations on the proliferation of (A) NCI-H1299 and (B) A549 cells

We treated lung cancer cells with different concentrations (0, 50, 100, 150, 200, and 250 µg/mL) of ginseng fat-soluble components. The results showed that the growth inhibitory effects of ginseng fat-soluble components on NCI-H1299 lung cancer cells were 19.62%, 32.80%, 44.28%, 60.22%, and 68.50%, respectively, compared with controls (Fig. 11; Table 3).

The IC50 concentration was further calculated to be 154.68 µg/mL. In subsequent experiments, NCI-H1299 cells were treated with 100 µg/mL ginseng fat-soluble components as the low-concentration group and 250 µg/mL ginseng fat-soluble components as the high-concentration group.

**Table 3:** Inhibitory effects of ginseng fat-soluble components at different concentrations on the proliferation of NCI-H1299 and A549 cells

	NCI-H1299		A549		
	Absorbance (450 nm)	Inhibition rate(%)		Absorbance (450 nm)	Inhibition rate(%)
A: control group	0.813 ± 0.063	0.00	0.798 ± 0.069		0.00
B: 50 µg/mL ginseng fat-soluble components group	0.654 ± 0.053	19.62	0.704 ± 0.050		13.43
C: 100 µg/mL ginseng fat-soluble components group	0.547 ± 0.040	32.80	0.343 ± 0.045		57.86
D: 150 µg/mL ginseng fat-soluble components group	0.453 ± 0.041	44.28	0.232 ± 0.019		71.48
E: 200 µg/mL ginseng fat-soluble components group	0.324 ± 0.016	60.22	0.187 ± 0.011		77.01
F: 250 µg/mL ginseng fat-soluble components group	0.256 ± 0.024	68.50	0.125 ± 0.020		84.58

### Effects of Ginseng Fat-soluble Components on Apoptosis in Lung Cancer Cells

**Fig. 12:**
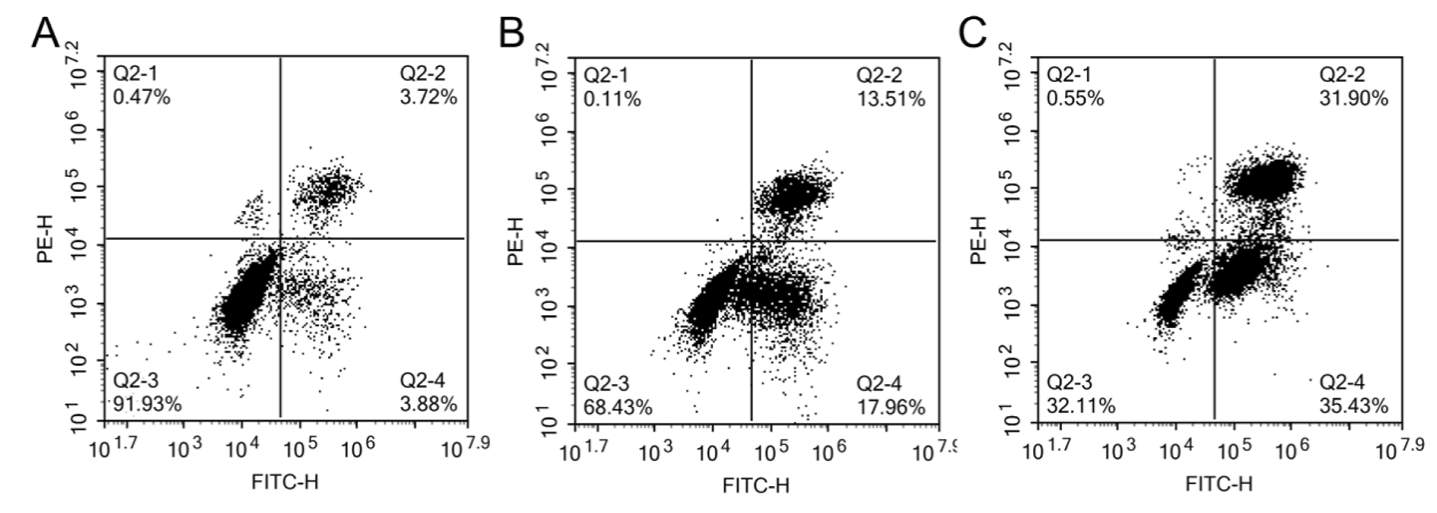
Effect of ginseng fat-soluble components on apoptosis in NCI-H1299 lung cancer cells; (A) control group; (B) 100 µg/mL treatment group; (C) 250 µg/mL group

**Fig. 13:**
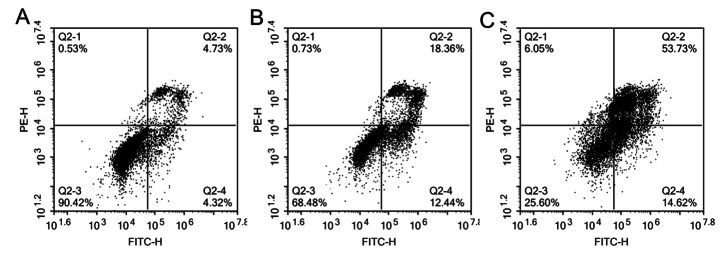
Effect of ginseng fat-soluble components on apoptosis in A549 lung cancer cells; (A) control group; (B) 100 µg/mL group; (C) 250 µg/mL group

Flow cytometry was used to analyze the effect of ginseng fat-soluble components on apoptosis in lung cancer cells. In NCI-H1299 cells, the apoptosis rates of Q2-2(%) + Q2-4(%) in the control group, low-concentration ginseng fat-soluble components group, and high-concentration ginseng fat-soluble components group were 7.6%, 31.47%, and 67.33%, respectively (Fig. 12). In A549 cells, the apoptosis rates of Q2-2(%) + Q2-4(%) in the control group, low-concentration ginseng fat-soluble components group, and high-concentration ginseng fat-soluble components group were 9.05%, 30.8%, and 68.35%, respectively (Fig. 13). Therefore, the fat-soluble components of ginseng promoted apoptosis in lung cancer cells (Table 4).

**Table 4:** Rate of ginseng fat-soluble components-induced apoptosis in NCI-H1299 and A549 cells

Groups	NCI-H1299 apoptosis rate(%)	A549 apoptosis rate(%)
Control	7.60	9.05
Ginseng fat-soluble components: low dose	31.47	30.80
Ginseng fat-soluble components: high dose	67.33	68.35

### Regulatory Effects of Ginseng Fat-soluble Components on Key Proteins in Lung Cancer Cells

**Fig. 14:**
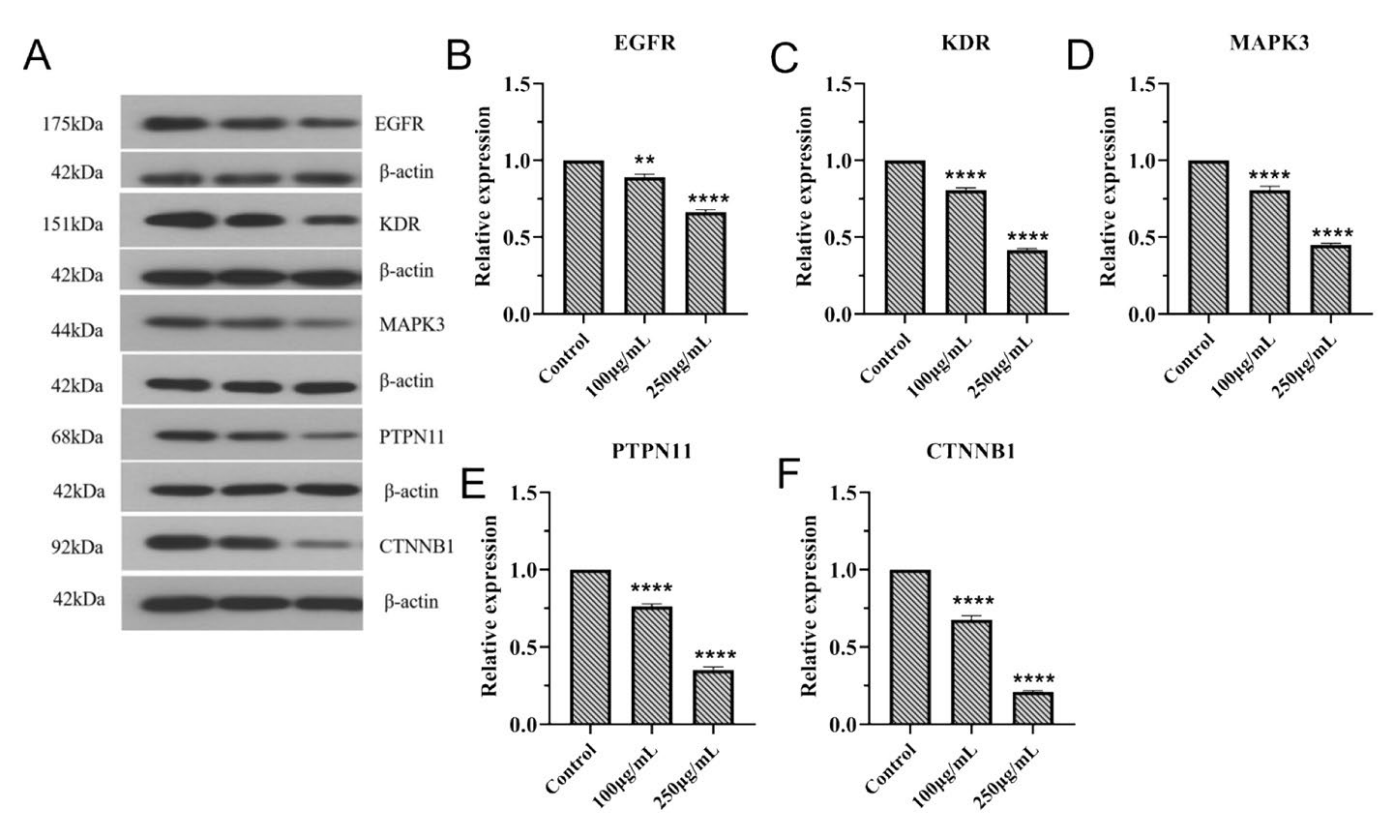
Effects of ginseng fat-soluble components on key proteins in NCI-H1299 lung cancer cells. (A) Western blot results. Graphs of relative (B) EGFR; (C) KDR; (D) MAPK3; (E) PTPN11; and (F) CTNNB1 expression; **P < 0.01; ***P < 0.001 vs. control

**Fig. 15:**
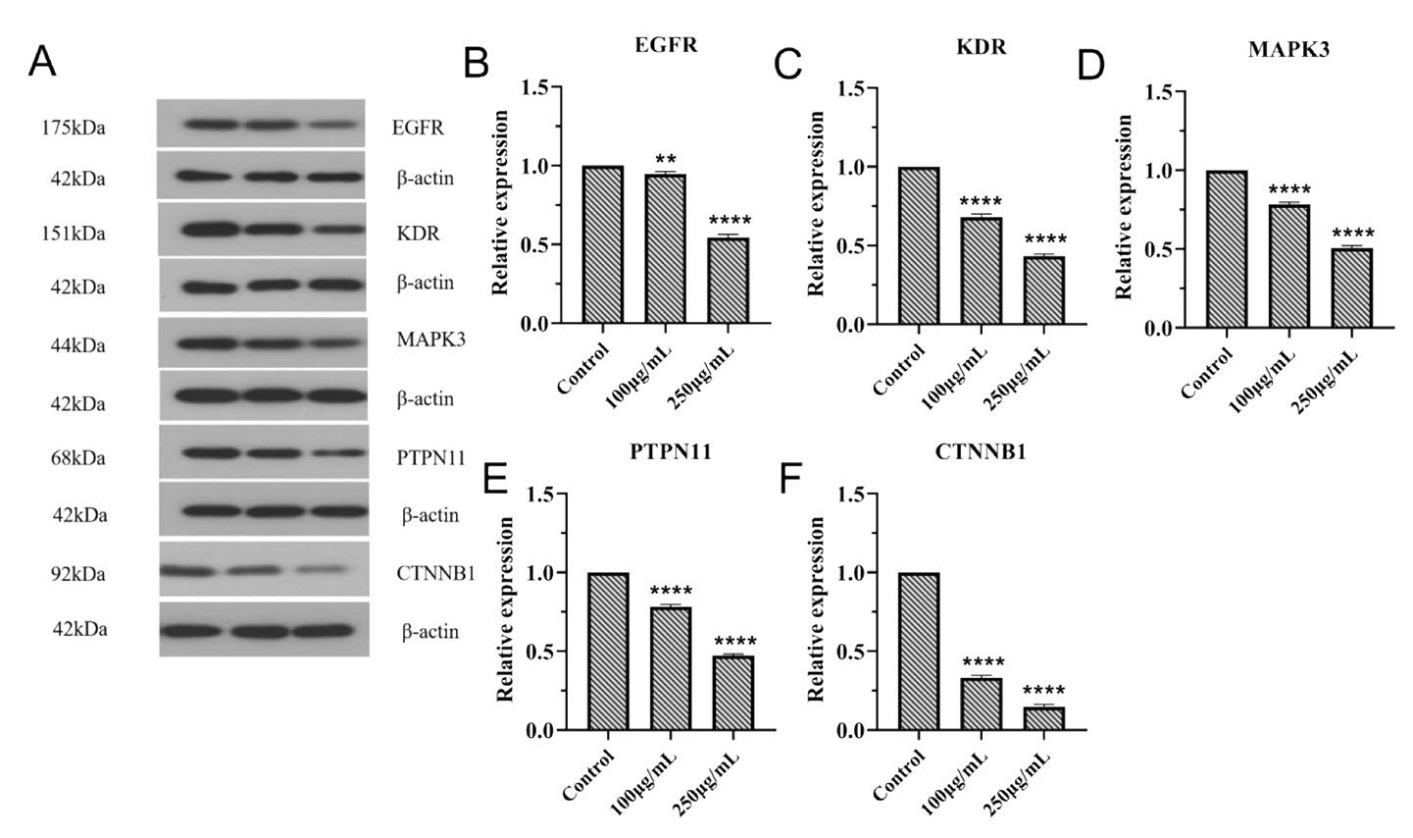
Effects of ginseng fat-soluble components on key proteins in A549 lung cancer cells. (A) Western blot results. Graphs of relative (B) EGFR; (C) KDR; (D) MAPK3; (E) PTPN11; and (F) CTNNB1 expression; **P < 0.01; ***P < 0.001 vs. control

Western blot analysis was used to detect the regulation of key proteins in lung cancer by ginseng fat-soluble components. The results of experiments in NCI-H1299 and A549 cells are shown in Figs. 14 and 15. Protein levels of EGFR, KDR, MAPK3, PTPN11, and CTNNB1 were significantly decreased in the ginseng fat-soluble components groups than in the control group (P < 0.05); additionally, levels of these proteins were significantly lower in the high-concentration group than in the low-concentration group (P < 0.05).

### Verification of mRNA Expression

**Fig. 16:**
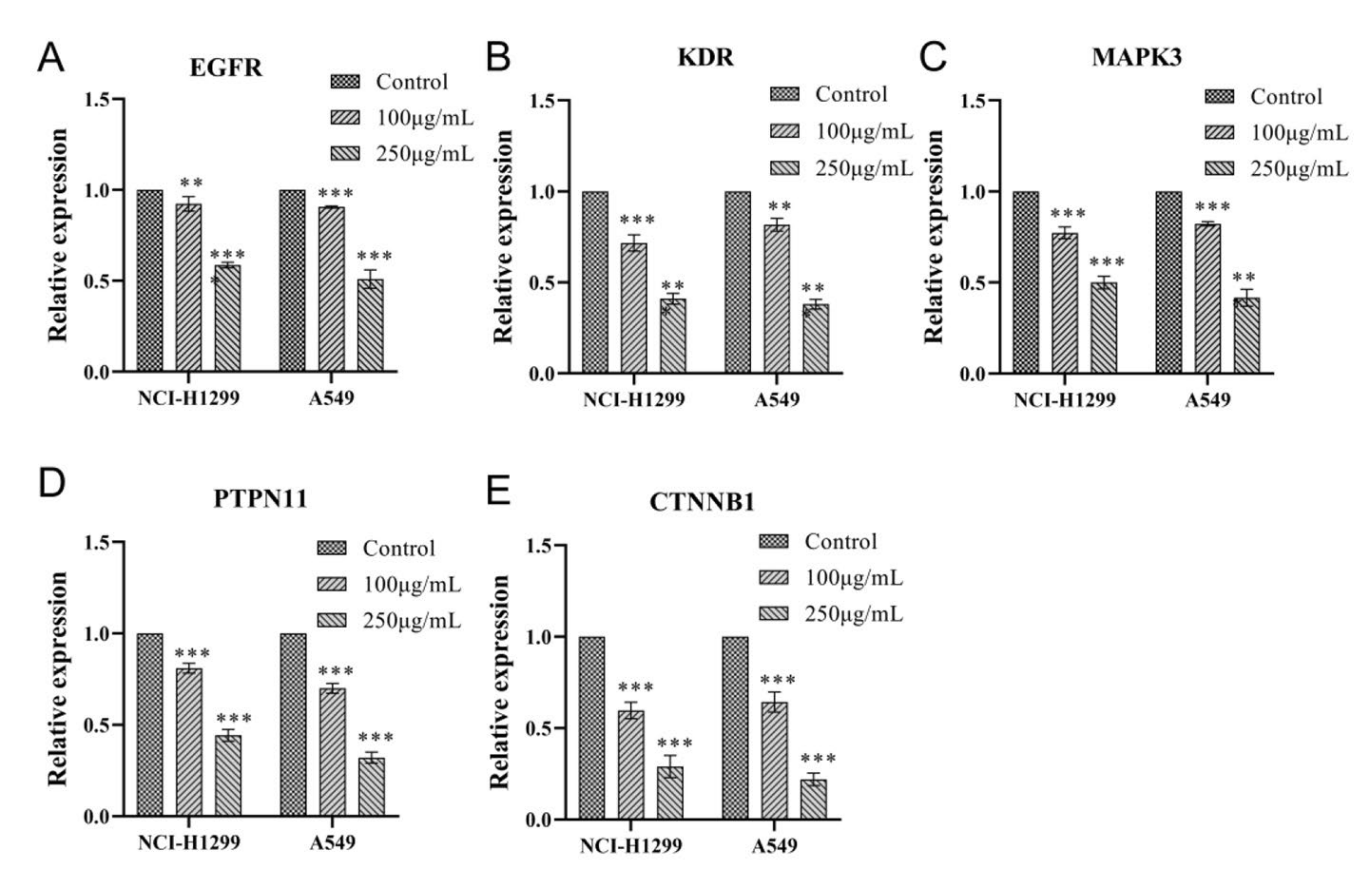
Regulation of the mRNA expression of key genes in lung cancer cells by ginseng fat-soluble components. Quantitative real-time PCR results for (A) EGFR; (B) KDR; (C) MAPK3; (D) PTPN11; and (E) CTNNB1; **P < 0.01; ***P < 0.001 vs. control

Verifications of mRNA expression levels are shown in Fig. 16. The mRNA levels of EGFR, KDR, MAPK3, PTPN11, and CTNNB1 were significantly decreased in the ginseng fat-soluble components groups compared with controls (all P < 0.05); furthermore, there were decreased levels of all five mRNAs in the high-concentration of fat-soluble components group compared with in the low-concentration group.

## Discussion

Lung cancer is one of the most common malignant tumors of the respiratory system and has the clinical characteristics of high invasiveness, early metastasis, and poor prognosis. Although some progress has been made in surgery-centered comprehensive treatment, the overall effect is not ideal. TCM is a unique method of treating malignant tumors in China. TCM not only focuses on the tumor itself but also pays attention to adjusting the overall state of the body, including the tumor microenvironment. An important mechanism of ginseng’s antitumor activity is also an important principle of TCM for treating malignant tumors—invigorating *qi* and supporting *zhengfa*. Strengthening the body and strengthening the foundation is the basic method of TCM for treating tumors. According to statistics, the most frequently prescribed medicines for treating tumors are tonic and nourishing medicines [9], of which the top one is the *qi*-tonifying medicine. Ginseng has the power of invigorating vitality and transforming body fluid. According to historical medical records, these treatments can adjust the middle, quench thirst, clear blood, and break the accumulation. Modern research has shown that ginseng Rg3 can induce apoptosis of human lung cancer A549 cells [10] and that the diol group of ginsenoside Rh2 inhibits Lewis cell proliferation [11]. Pan et al. [12] reported that ginseng water extract can effectively inhibit the growth of lung squamous cell carcinoma cells induced by N-nitroso-trischloroethyl-urea, with the inhibition rate reaching 65%. Zhang et al. [13] found that both S-type and R-type ginsenoside Rh2 can inhibit human lung adenocarcinoma A549 cells. The proliferation of lung adenocarcinoma A549 cells was inhibited by 25 mg/mL S-type and R-type ginsenoside Rh2 after a 48 h incubation (33.6% and 28.5%, respectively). Wong et al. [14] inoculated lung cancer cells into nude mice and found that ginseng alcohol extract significantly slowed the growth of lung tumor xenografts in nude mice.

In this study, the main fat-soluble components of ginseng were identified by GC-MS. Combined with the TCMSP database, the main active fat-soluble components of ginseng were determined to be linoleic acid, palmitoleic acid, oleic acid, bocacene, (+)-bucalene, ginseng epoxy alkynol, methyl 8, 11–18 dienoate, phenyl-beta-glucopyranoside, cineolenol, and ginseng alkynol. Among them, there are many reports that ginseng alkynol has inhibitory effects on the proliferation of a variety of tumor cells [15–18]. Ginsenthinyl alcohol has cellular activity and can inhibit the synthesis of DNA, RNA, and protein in latent lympho-leukemia L1210 cells. The cellular activity of ginsenthinyl alcohol is related to the chemical composition of C-9 and C-10 in its structure and it can inhibit cell proliferation by regulating the cell cycle. The molecular mechanism is via increased expression of the cyclin-dependent kinase inhibitors P27kip1 and P21waf1 and decreased expression of cyclin 2, which leads to cell growth stagnation in G1 phase. The malignancy of hL-60 cells was significantly reduced by treatment with ginsenynol, and the cells differentiated into monocytes/macrophages. The induced differentiation was related to activation of intracellular protein kinase C and cAMP signaling. Polyacetylene alcohol compounds contained in ginseng have significant anti-inflammatory, anti-platelet aggregation, inhibition of cell oxidase, inhibition of leukemia growth, and inhibition of thrombosis effects. Low dose of ginseng epoxynol can effectively induce the differentiation of hepatocellular carcinoma HepG2 and SMMC-7721 cells into normal cells [19].

We used network pharmacology combined with bioinformatics to screen out 33 common targets of ginseng fat-soluble components in lung cancer. GO and KEGG pathway analysis of the targets showed that the GO enrichment of core genes mainly focused on the synthesis and transcriptional regulation of genetic materials such as membrane rafts, nucleotide metabolism, production of positive regulatory cytokines, peptide tyrosine autophosphorylation, and protein transport regulation. GO enrichment also found the reaction of cells to nitrogen compounds, reaction to hormones, positive regulation of response to external stimulation, reaction to epidermal growth factor, and reaction to heterogeneous stimulation. These basic biological processes and functions are reflected in the regulation of target genes and specific pathways by the active components of ginseng fat-soluble components. Regarding KEGG pathway enrichment, it mainly included VEGF signaling, regulation of lipolysis in adipocytes, chronic myeloid leukemia, endocrine resistance, and NSCLC. These pathways play important regulatory roles in NSCLC. Additionally, VEGF protein is associated with local metastasis and prognosis in NSCLC [20].

We further obtained the core nodes of the PPI network by constructing the PPI network of key target genes and using Cytoscape to further construct TCM-component-disease-target and TCM-component-pathway-target networks. For subsequent experimental verification, the top 10 targets among the 33 key targets (EGFR, KDR, MAPK3, PTPN11, CTNNB1, NR3C1, PPAGR, AKT1, SP1, and MAPK14) were reduced to five by literature mining, so that EGFR, KDR, MAPK3, PTPN11, and CTNNB1 were used for further molecular experiments. The experimental results showed that ginseng fat-soluble components negatively regulated EGFR, KDR, MAPK3, PTPN11, CTNNB1 protein levels in lung cancer NCI-H1299 and A549 cells; moreover, the protein expression in the high-concentration treatment group was significantly lower than in the low-concentration group. We further verified the mRNA levels by qPCR and found that mRNA expression of EGFR, KDR, MAPK3, PTPN11, and CTNNB1 were significantly lower in the ginseng fat-soluble components groups than in the control group, with high concentration of the fat-soluble components having a stronger inhibitory effect than low-concentration treatment.

EGFR is recognized to be closely related to the occurrence of lung cancer, and epidermal growth factor tyrosine kinase inhibitors (EGFR-TKIs) have been widely used to treat EGFR-mutant NSCLC. VEGF is a key ligand that promotes angiogenesis and mainly promotes the formation of vascular endothelium and increased vascular permeability. VEGF can produce biological effects by binding to its receptor, which is a protein tyrosine kinase that is expressed on human vascular endothelial cells and most tumor cells. Among them, fetal liver kinase-1 contains a kinase insert functional domain receptor, among which the FLK-1/KDR (kinase insert domain-containing receptor, KDR) is the most important.

Angiogenesis in lung cancer tissues not only depends on the amount of VEGF secretion, but also requires a series of signal transduction events downstream of receptor binding, namely FLT-1 and KDR. Both FLT-1 and KDR are expressed on lung cancer cells, and previous experiments have confirmed that VEGF can stimulate KDR expression; moreover, only the KDR expression by tumor cells is consistent with VEGF expression, which is consistent with the results of this study and suggests that angiogenesis may be primarily mediated by KDR. Studies have shown that EGFR mutations occur in 39–81% of NSCLC patients, which is basically consistent with carcinoembryonic antigen [21–23]. EGFR is a member of the ErbB family of receptor tyrosine kinases. EGFR mutations can continuously activate downstream pathways and promote proliferation, survival, invasion, and angiogenesis [24]. The PI3K/Akt pathway is one of the main downstream signaling pathways of EGFR. When EGFR is mutated, the PI3K/Akt pathway is continuously activated, accelerating NSCLC progression [25].

Studies have shown that EGFR mutations can also upregulate the expression of programmed death receptor-1 (PD-1) [26, 27], programmed death receptor-ligand 1 (PD-L1), and cytotoxic T lymphocyte antigen 4 proteins by activating downstream MAPK signaling, which ultimately leads to immune escape. PTPN11 is required for activation of the RAS/ERK pathway by most receptor tyrosine kinases and may provide a common node of resistance. Studies have found that the combination of the PTPN11 inhibitor SHP099 and MEK inhibition can suppress the proliferation of various cancer cells including lung cancer; similarly, PTPN11 knockout and MEK inhibition have similar effects in vitro [28].

CTNNB1 encodes the intracellular scaffold protein β-catenin. On the basis of PPIs, β-catenin is involved in cell-cell adhesion, signal transduction, and transcriptional regulation. Studies have shown that dysregulated β-catenin signaling is involved in chronic inflammation, organ fibrosis, and various types of human cancers [29]. Missense mutations or in-frame deletions near S33, S37, T41, and S45 in β-catenin lead to functionally acquired β-catenin mutants that are resistant to ubiquitin-mediated proteasome degradation and induce the upregulation of oncogenic target genes such as CCND1 and MYC in tumors, including lung cancer. Abnormal β-catenin-dependent transcriptional activation drives human carcinogenesis by inducing cancer stem cell characteristics, tumor cell proliferation, and epithelial-to-mesenchymal transformation in solid tumors [30].

In this study, a drug-component-target-disease network was constructed through the network pharmacology method to explore the active components, potential therapeutic targets, and mechanisms of ginseng fat-soluble components. Furthermore, we elaborated on our understanding of the role of ginseng fat-soluble components in the treatment of lung cancer. Subsequent experiments verified the effect of ginseng fat-soluble components on the expression of EGFR, KDR, MAPK3, PTPN11 and CTNNB1, which preliminarily indicated that ginseng fat-soluble components may affect the regulation of related pathways by down-regulating these five key proteins and thus may have an impact on the treatment of lung cancer.

## Data Availability

The datasets generated and/or analyzed during the current study are available from the corresponding author upon reasonable request.
